# The integration of machine learning and multi-omics analysis provides a powerful approach to screen aging-related genes and predict prognosis and immunotherapy efficacy in hepatocellular carcinoma

**DOI:** 10.18632/aging.204876

**Published:** 2023-07-28

**Authors:** Jiahui Shen, Han Gao, Bowen Li, Yan Huang, Yinfang Shi

**Affiliations:** 1 Department of Pharmacy, Huzhou Maternity and Child Health Care Hospital, Huzhou, China; 2 Department of Stomatology, First Affiliated Hospital of Huzhou University, Huzhou, China; 3 School of Pharmacy, Anhui Medical University, Hefei, China

**Keywords:** machine learning, aging, hepatocellular carcinoma, prognostic model, immunotherapy

## Abstract

Background: Hepatocellular carcinoma (HCC) is a highly malignant tumor with high incidence and mortality rates. Aging-related genes are closely related to the occurrence and development of cancer. Therefore, it is of great significance to evaluate the prognosis of HCC patients by constructing a model based on aging-related genes.

Method: Non-negative matrix factorization (NMF) clustering analysis was used to cluster the samples. The correlation between the risk score and immune cells, immune checkpoints, and Mismatch Repair (MMR) was evaluated through Spearman correlation test. Real Time Quantitative PCR (RT-qPCR) and immunohistochemistry were used to validate the expression levels of key genes in tissue and cells for the constructed model.

Result: By performing NMF clustering, we were able to effectively group the liver cancer samples into two distinct clusters. Considering the potential correlation between aging-related genes and the prognosis of liver cancer patients, we used aging-related genes to construct a prognostic model. Spearman correlation analysis showed that the model risk score was closely related to MMR and immune checkpoint expression. Drug sensitivity analysis also provided guidance for the clinical use of chemotherapy drugs. RT-qPCR showed that TFDP1, NDRG1, and FXR1 were expressed at higher levels in different liver cancer cell lines compared to normal liver cells.

Conclusion: In summary, we have developed an aging-related model to predict the prognosis of hepatocellular carcinoma and guide clinical drug treatment for different patients.

## INTRODUCTION

Hepatocellular carcinoma is a highly malignant tumor and is one of the leading causes of cancer-related deaths worldwide [1]. Despite significant progress in the diagnosis and treatment of HCC, its incidence and mortality rates remain high [2]. In clinical practice, early diagnosis and treatment of HCC are of paramount importance. Numerous studies have shown that early detection and intervention can greatly improve patient prognosis [3, 4]. Therefore, finding new methods for the diagnosis and treatment of HCC is currently a hot topic in HCC research. In recent years, extensive genomic and transcriptomic studies have shown that aberrant changes in many genes and signaling pathways may contribute to HCC formation and progression [5–7]. These research findings provide new ideas and methods for the diagnosis and treatment of HCC. Traditional treatment methods for HCC include surgical resection, radiation therapy and chemotherapy, but these treatments still have many problems and limitations [8, 9]. With the emergence of new technologies and drugs, personalized therapy is gradually becoming a new trend in HCC treatment [10]. For example, targeted therapy against tumor-related signaling pathways has become a hot topic in HCC treatment [11]. In addition, immunotherapy as a novel approach to HCC treatment has also received widespread attention [12, 13]. In summary, HCC is a serious disease. Due to the complex etiology and mechanisms of HCC, the therapeutic effects of HCC vary greatly. Therefore, new biomarkers and prognostic models are needed to achieve precision management for individuals.

Aging with the significant feature of permanent growth arrest is often a response to endogenous and exogenous stresses, including telomere dysfunction, oncogene activation, and persistent DNA damage [14]. The generation of senescent cells occurs throughout a person’s life and plays a functional role in various physiological and pathological processes, including embryonic development, wound healing, host defense, and tumor suppression [15]. Studies have shown that aging is an effective barrier to prevent tumor development [16]. Cell senescence is associated with the decline of hematopoietic stem cell (HSC) function and an increased risk of malignancies in the hematopoietic system, especially leukemias, multiple myeloma, myelodysplastic syndromes, and lymphomas, which are more common in the elderly [14]. According to the literature, cell senescence can promote skin carcinogenesis through the p38MAPK and p44/42MAPK signaling pathways [17]. Additionally, research has found that the aging-related SIN3B can promote inflammation and pancreatic cancer progression [18]. Liu et al. research has shown that dysbiosis of the liver microbiota can cause activation and aging of hepatic stellate cells, thereby driving the progression of liver cirrhosis to hepatocellular carcinoma [19]. Previous research has revealed a close relationship between aging and cancer. However, current studies on the link between liver cancer and aging are often limited to individual molecules, and research on multiple key aging genes and liver cancer is still lacking.

The aim of this study is to construct a risk prognosis model by integrating multiple key genes related to aging and to explore the relationship between risk score and immune cell and tumor microenvironment by combining bulk and single-cell sequencing. Furthermore, we investigate their correlation with MMR, immune checkpoints, and IC50 to determine the effectiveness of immune therapy and different chemotherapy drugs. In addition, we validated these results by multi-omics analysis and basic experiments.

## MATERIALS AND METHODS

### Data source

Transcriptomic and clinical data for hepatocellular carcinoma were downloaded from The Cancer Genome Atlas (TCGA) (https://portal.gdc.cancer.gov/) database, including 374 cancer samples and 50 normal samples. Clinical data included survival status, survival time, gender, grade, and TNM stage. The liver cancer-related dataset (GSE14520) was downloaded from the GEO database (https://www.ncbi.nlm.nih.gov/), and after collating the data, a total of 221 liver cancer samples were used for subsequent model verification analysis. Dataset GSE39791 was used for differential analysis of cancerous and paraneoplastic tissues.

### NMF clustering analysis

Non-Negative Matrix Factorization (NMF) is a commonly used clustering method for subgroup identification. Prior to the clustering analysis, the data were filtered and sorted. Subsequently, the differential analysis and prognosis of 278 aging-related genes were performed, followed by the application of the NMF algorithm to classify the samples into two clusters, namely C1 and C2. The C1 cluster consisted of 68 samples, while the C2 cluster consisted of 153 samples.

### Functional enrichment analysis

To explore the underlying biological processes and signaling pathways associated with the acquisition of differential genes, gene ontology (GO) and KEGG enrichment analyses were performed using the “clusterProfiler” R package. GO analysis included BP, CC and MF. The annotated gene set file is “c2.cp.kegg.v7.4.symbols.gmt” and “c5.go.v7.4. symbols.gmt”. A threshold value of *P* value < 0.05 was determined.

### Single-cell data analysis

Single cell sequencing data were obtained from the GEO database (GSE146115). The Seurat package analyzes the dataset and clusters the samples after PCA dimensionality reduction and t-SNE dimensionality reduction. The SingleR package was used for cell type annotation of single-cell data.

### Weighted gene co-expression network analysis

WGCNA is an analytical method for analyzing gene expression patterns of multiple samples. Genes with similar expression patterns can be clustered and the association between modules and specific traits or phenotypes can be analyzed. Aging-associated genes were used to construct the weighted correlation network, where prognosis-related modules were selected for further analysis.

### Modeling analysis

A risk score model was constructed using the screened key genes, and a risk score was also calculated for each patient. A comprehensive evaluation of the role of these key molecules in the prognosis of patients with hepatocellular carcinoma. Lasso regression analysis was used to construct a prognostic model. The risk score of each HCC patient was calculated by the formula: risk score = (Expression of FXR1 × coefficient) + (Expression of NDRG1 × coefficient) + (Expression of TFDP1 × coefficient). The TCGA dataset is divided into training and test sets, while we use the GSE14520 dataset for further model validation. Survival analysis was performed using KM curves to assess whether there was a difference in survival between high and low risk groups of patients with liver cancer in the training and test sets. Subsequently, risk-survival curves were used to assess patient survival and death in the high and low risk groups and how the key genes that were modeled differed between the two groups. And the ROC curve is used to determine the effectiveness of this prediction model.

### Immunoassay and drug sensitivity analysis

The Cibersort algorithm was used to quantify the abundance of various immune cells in each sample. In total, we evaluated 22 human immune cells. The immune score, stromal score and total score are derived by the ESTIMATE algorithm to assess the immune microenvironment in the tumor tissue. The tumor stemness index is an important metric used to assess the similarity between tumor cells and stem cells. Analyzing the correlation between the stemness index and the risk score can help predict whether the model can serve as an indicator of stemness. Correlations with immune checkpoints and MMR were calculated using Spearman correlation analysis to determine the suitability of hub genes for predicting the efficacy of immunotherapy. Immunotherapy was validated between different risk groups using the IMvigor210 dataset. The “oncoPredict” package was used to assess drug sensitivity in different groups, and we analyzed the current chemotherapeutic agents that have some relevance to liver cancer.

### Real-Time quantitative PCR (RT-qPCR)

Total RNA was extracted using TRIZOL (#15596026, Invitrogen, USA), and the concentration was determined and placed on ice for use. Add the calculated amount of RNA to PCR tubes, generally reverse transcribe 500 ng of total RNA per tube, add PrimeScript™ RT Master Mix (Takara Bio, Japan) about 2 ul, and add RNase free water (Takara Bio, Japan) to fix the volume to 10 μl. Set up the reaction program: 37°C for 15 min, 85°C for 5 s. Reverse transcription was completed to obtain cDNA. Dilute the qPCR primers to 10 μM and mix the Forward and Reverse primers in equal volumes. Prepare the qPCR system in the following proportions: 2xTB Green (#RR820L, Takara, Japan) 10 μl, ddH2O 8 ul, template cDNA 1 μl, primers (Forward primer + Reverse primer) 1 μl. Run the qPCR according to the following procedure: 94°C for 2 min, 94°C for 30 s, 60°C for 32 s, 60–94°C for 45 cycles, and collect the solubility curve. Data were collected and qPCR results were analyzed [20]. The primer sequences are as follows: FXR1-F: 5′-GAGAAGACGGTATGGTTCCATTT-3′, FXR1-R: 5′-AGGCGTTCCATTCTTAGCTGT-3′; NDRG1-F: 5′-CTCCTGCAAGAGTTTGATGTCC-3′, NDRG1-R: 5′-TCATGCCGATGTCATGGTAGG-3′; TFDP1-F: 5′-AATTGAAGCCAACGGAGAACTC-3′, TFDP1-R: 5′-CGGTCTCTGAGGCGTACCA-3′; GAPDH-F: 5′-GGAGCGAGATCCCTCCAAAAT-3′, GAPDH-R: 5′-GGCTGTTGTCATACTTCTCATGG-3′.

### Immunohistochemistry analysis

The Human Protein Atlas (HPA) (https://www.proteinatlas.org/) database is based on proteomic, transcriptomic and systems biology data for statistical analysis, and covers protein expression in normal and tumor tissues. Among them, the expression of FXR1 and NDRG1 in the liver, which we used to construct aging-related prognostic models, was also included.

### Data statistics

The Wilcoxon test was used for the analysis of differences between the two groups, while the correlation analysis was based on the Spearman correlation test. Kaplan-Meier analysis and log-rank test were used to compare the survival analysis between the two groups. *P* values are bilateral and *P* < 0.05 is considered statistically significant. R software (version 4.2.2) was used to perform the statistical analysis.

### Data availability statement

The article/supplementary material contains the original contributions presented in this study. For further information, please contact the corresponding author.

## RESULTS

### NMF clustering analysis

**Figure 1:**
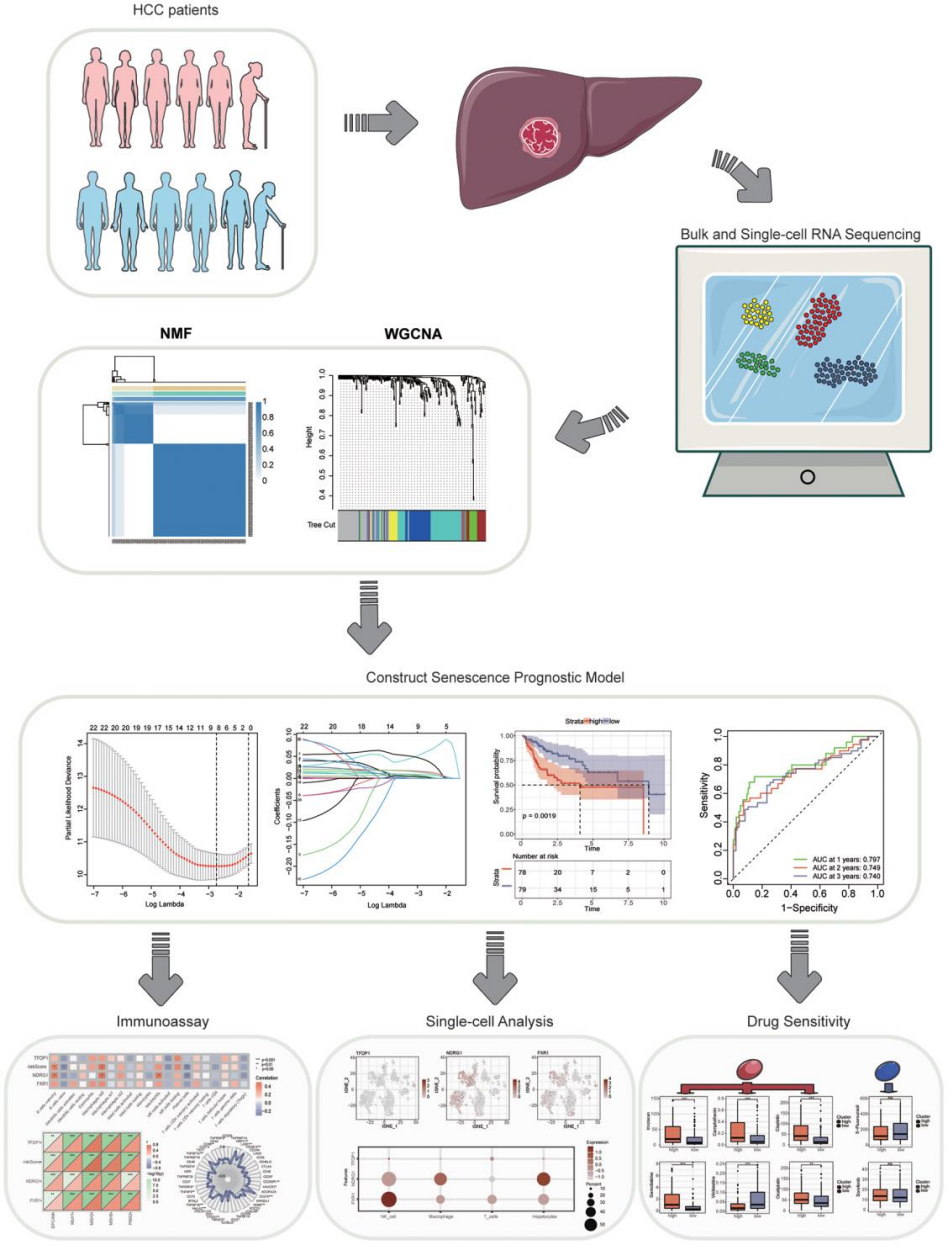
A flow chart of the manuscript.

**Figure 2:**
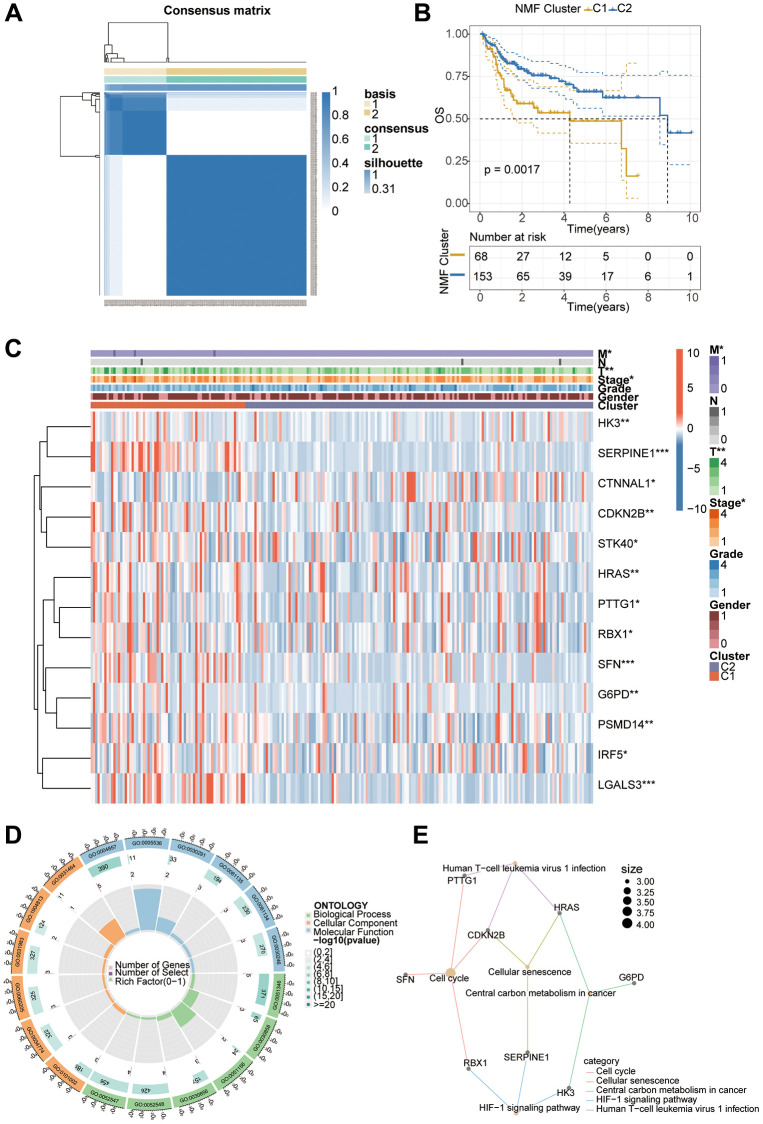
**NMF staging and correlation analysis of patients with hepatocellular carcinoma.** (**A**) Patients were classified into two clusters using the NMF algorithm. (**B**) Prognostic analysis revealed a poorer prognosis for patients with the C1 cluster. (**C**) Exploration of differential genes between different subtypes by differential analysis. (**D**, **E**) GO and KEGG analysis to investigate the underlying mechanisms and pathways.

First, we draw a flowchart illustrating the whole analysis process in detail (Figure 1). As mentioned previously, we obtained RNA-Seq data and relevant clinical information for hepatocellular carcinoma through the TCGA database. A total of 278 aging-associated genes were obtained from the CellAge database (https://genomics.senescence.info/cells/), and we performed NMF Clustering based on the expression matrix of these aging-associated genes. It can be seen that the samples are better divided into two clusters (Figure 2A). KM analysis was used to analyze the prognostic differences between the two groups of patients, and we saw a poorer prognosis for patients in the C1 cluster (Figure 2B). Immediately after, we performed a differential analysis of the two clusters of samples and Hierarchical clustering clearly showed a total of 13 aging-related differential genes (*p* < 0.05). Also, we found significant differences in T and M staging and Stage between the two clusters (Figure 2C). Subsequently, we performed GO analysis. BP was mainly enriched in regulation of epithelial cell differentiation, CC was mainly enriched in vesicle lumen and secretory granule lumen, while MF was mainly enriched in protein serine/threonine kinase inhibitor activity (Figure 2D). And the main pathways enriched by KEGG analysis are cell cycle, cellular senescence, central carbon metabolism in cancer, and HIF-1 signaling pathway (Figure 2E).

### Single-cell data analysis

**Figure 3:**
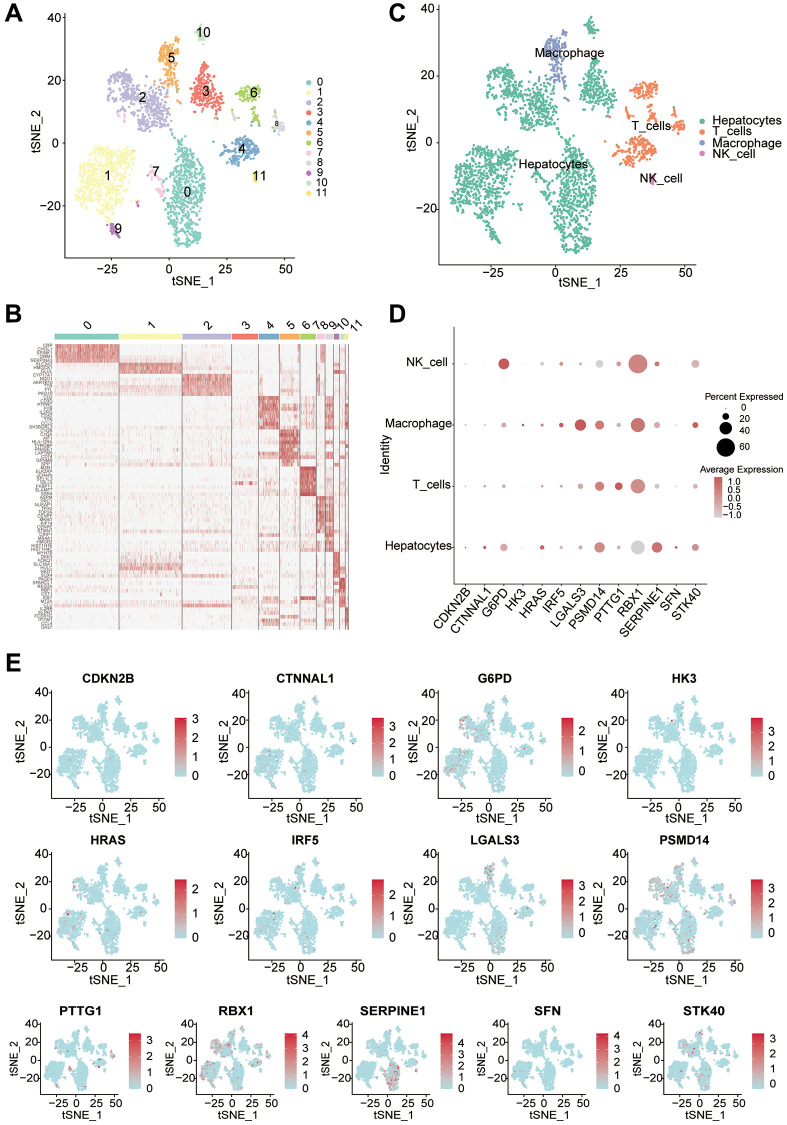
**Single-cell analysis reveals the expression of differential genes in different cell types.** (**A**) Using the tSNE algorithm to dimensionalize the samples into 12 clusters. (**B**) Heat map clearly showing the major differentially expressed genes in different clusters. (**C**) The Seurat package annotates different clusters with a total of 4 classes of cells. (**D**) The bubble diagram shows the expression of difference genes in different cells. (**E**) Visualization of 13 differential genes by single-cell sequencing.

We downloaded single-cell sequencing data (GSE146115) from the GEO database for liver cancer tissues, and collated the data for a total of 3200 single-cell comprehensive transcriptional profiles. The PCA and tSNE dimensionality reduction analysis of the samples allowed us to divide the samples into 12 clusters, and the subsequent heat map shows the differential genes between the different clusters (Figure 3A, 3B). We then annotated the 12 clusters of cells and could find that the cells were clearly divided into four classes. In addition to the main component hepatocellular carcinoma cells, there are macrophages, T cells and NK cells (Figure 3C). Subsequently, the expression of typing difference genes between different cells in liver cancer tissues was analyzed. The scatter plot clearly shows that RBX1 is expressed in the highest amount in all cells. In addition, the expression of RBX1 was again significantly higher in NK cells than in other cells (Figure 3D, 3E).

### Immunoassay of NMF clustering

**Figure 4:**
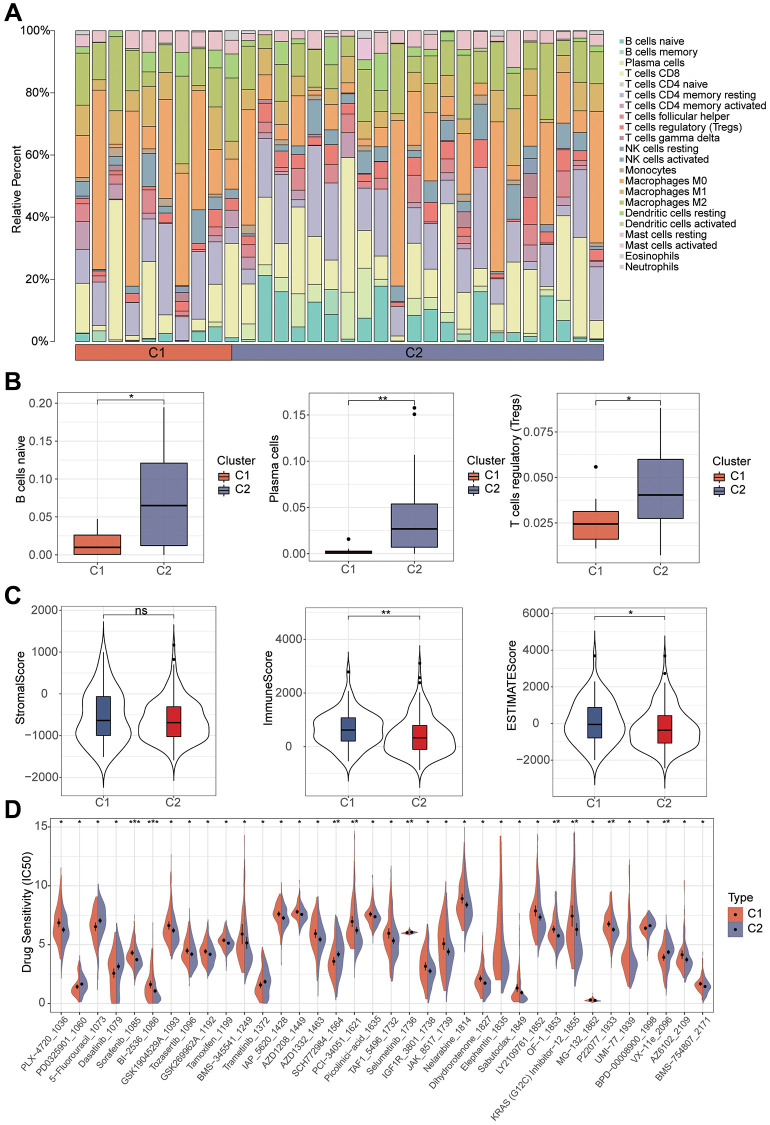
**Immunoassay and drug sensitivity analysis of samples with different cluster.** (**A**) The histogram shows the expression of immune cells between the different typologies. (**B**) Expression of B cell naive, Plasma cell and Tregs between different clusters. (**C**) Exploring the correlation of different clusters with the tumor microenvironment. (**D**) Analysis of the differences between the various chemotherapeutic agents in the different clusters.

To explore the immune cell landscape between different NMF clusters, we performed the analysis of immune infiltration by the Cibersort algorithm. First, we can see how the various types of immune cells are different between the two clusters. Among them, B cell naive, plasma cells, and Tregs had significant differences between the two types, and the expression in C2 clusters was higher than that in C1 cluster (Figure 4A, 4B). The estimate algorithm allowed exploring the differences in immune scores, stromal scores and total scores between the two clusters, and we found that immune scores were significantly different between the two clusters (Figure 4C). We then investigated whether there were differences in drug sensitivity of chemotherapeutic drugs between the two clusters and plotted graphs for those indicators that were significant (Figure 4D).

### Analysis of prognostic models

**Figure 5:**
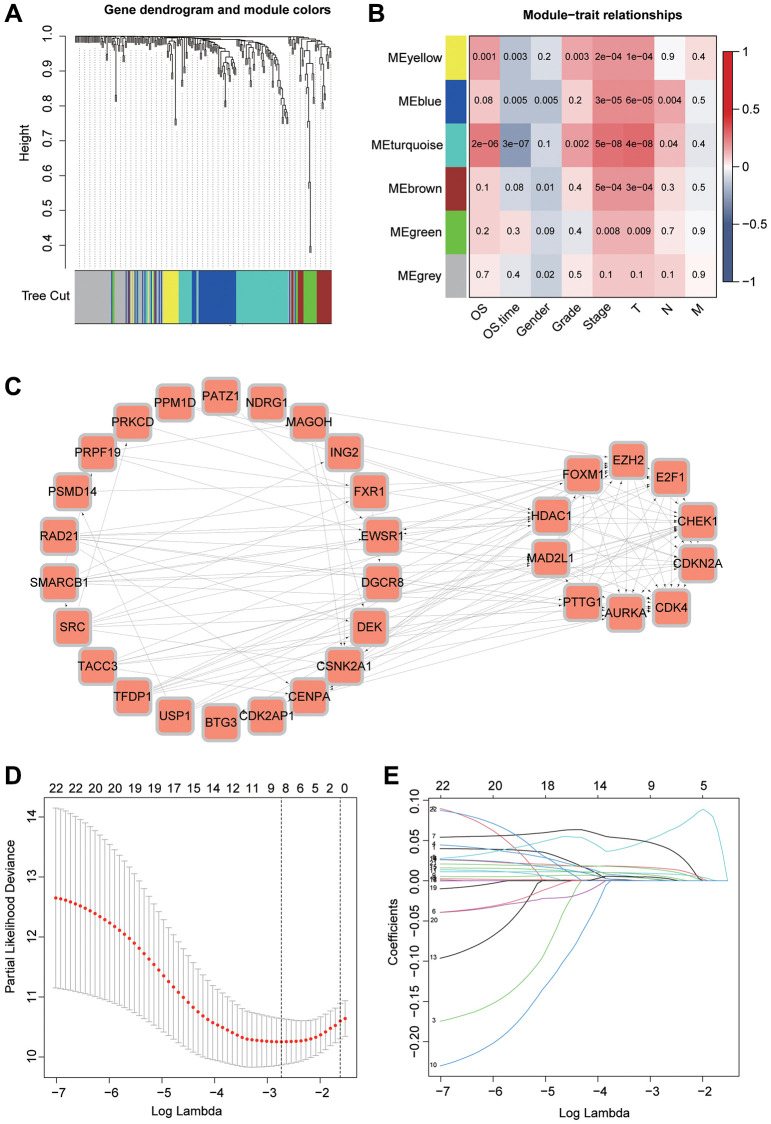
**WGCNA combined with Lasso algorithm to construct a prognostic model.** (**A**) Clustering of genes using the WGCNA algorithm. (**B**) Clinical and prognostic analysis of the genes in different modules. (**C**) Study of associations between genes of the MEturquoise module using the STRING database. (**D**, **E**) Selection of valuable genes by Lasso algorithm to construct prognostic models.

**Figure 6:**
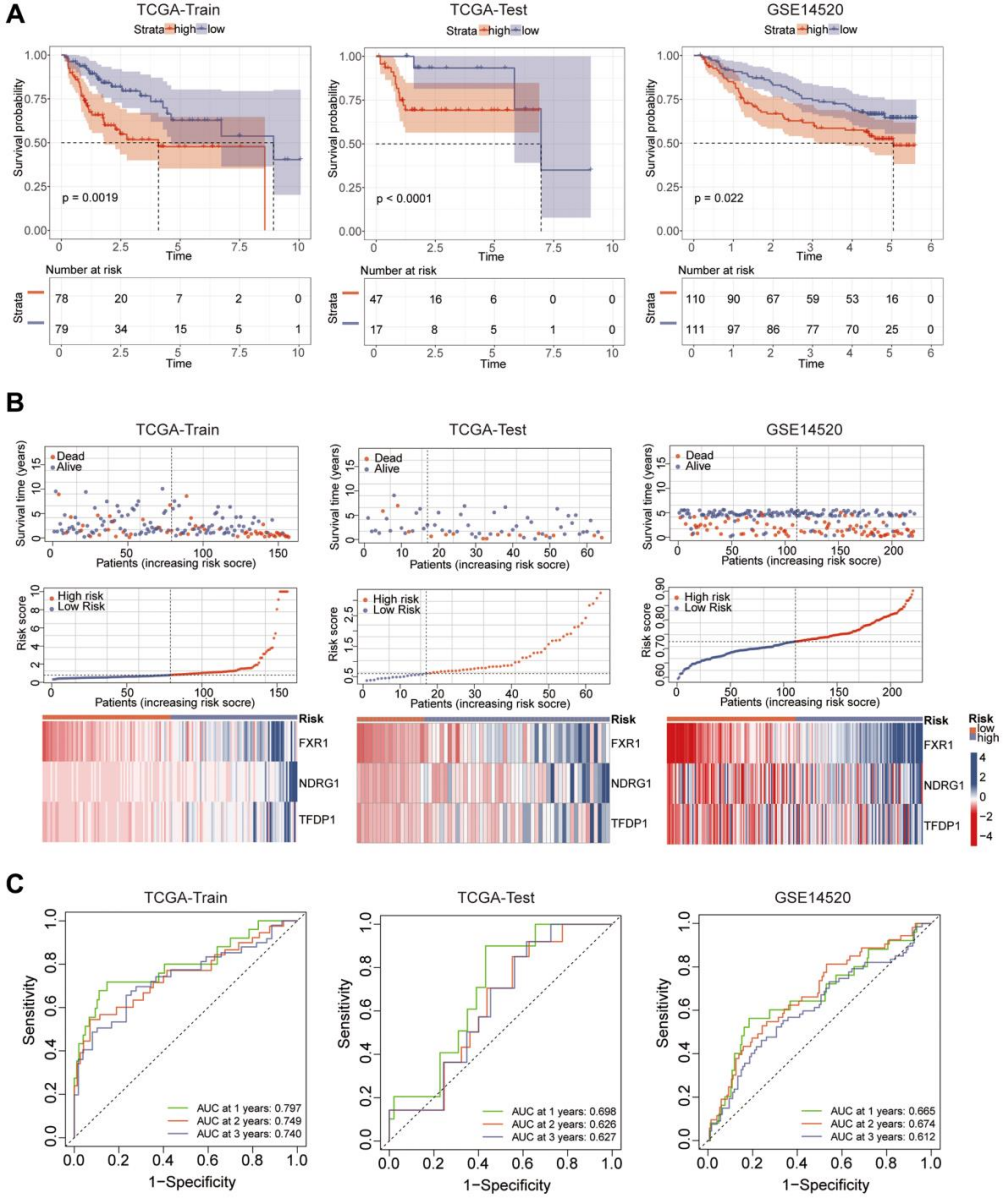
**Prognostic analysis and model efficacy validation.** (**A**) Prognosis between high and low risk groups was analyzed by KM curves. (**B**) Risk curves showing the differences between high and low risk groups. (**C**) ROC curves analyzing the specific efficacy of the model.

Considering that aging-related genes may be highly correlated with the prognosis of hepatocellular carcinoma patients, we used these genes to construct a prognostic model. First, we clustered the aging genes by WGCNA, and we could see that the genes were divided into a total of 6 modules. Based on the *p*-values, we observed that the MEturquoise module exhibits the highest correlation with patient prognosis (Figure 5A, 5B). For differential and prognostic analysis of aging-related genes in this module, we screened a total of 38 hub genes. Through the string database, we explored the correlation between these genes (Figure 5C). We identified FXR1, NDRG1 and TFDP1 to construct the model by lasso analysis. The risks core of each sample was calculated according to Equation (Figure 5D, 5E). We validated the model using 3 datasets considering the correlation between this risk score and patient prognosis. The TCGA dataset is first divided into training and validation sets using the R package “caret” while GSE14520 is also used as the validation set. It can be seen that patients with high-risk scores in all three datasets have a poor prognosis (Figure 6A). To further investigate the survival status of both groups, we found that patients in the high-risk group had a higher mortality rate. And the heat map revealed that the expression of all three key genes used for modeling were significantly higher in the high-risk group than in the low-risk group (Figure 6B). ROC curves can be seen for the TCGA training set model with AUC values of 0.797, 0.749 and 0.740 for 1, 2 and 3 years While the validation set AUC values are 0.698, 0.626, and 0.627 respectively. Finally, the AUC values for GSE14520 were 0.665, 0.674, and 0.612 (Figure 6C).

### Prognostic model immune landscapes drug sensitivity analysis

**Figure 7:**
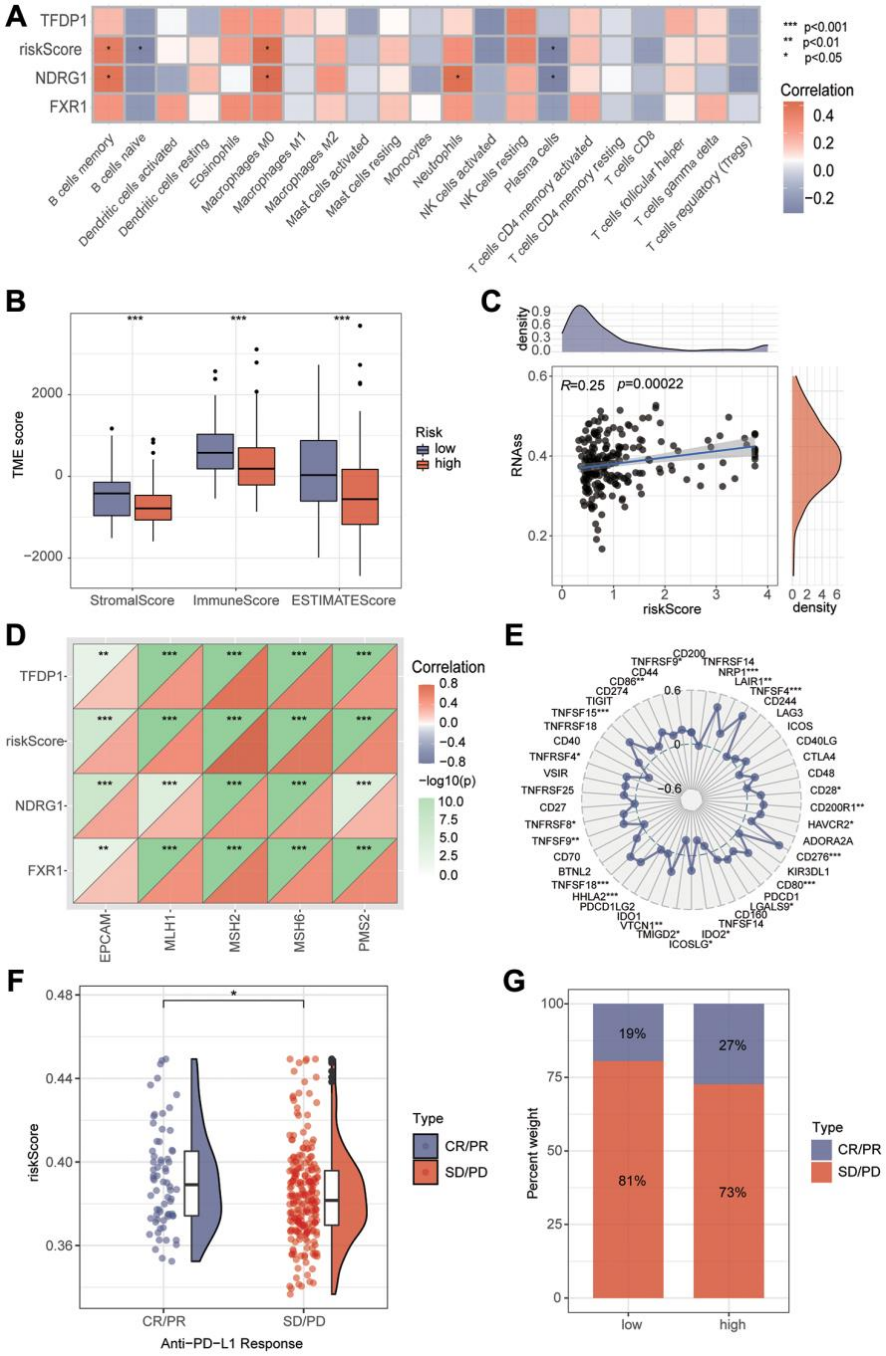
**Immunological analysis between high and low risk groups.** (**A**) Correlations between risk scores and different immune cells were calculated by the Cibersort algorithm. (**B**) Tumor microenvironment analysis to assess the differences between high and low risk groups. (**C**) Tumor stemness analysis found that risk scores were strongly correlated with tumor stemness. (**D**) Correlation analysis found that risk scores were strongly correlated with MMR. (**E**) Correlation analysis revealed that risk scores were strongly correlated with immune checkpoints. (**F**, **G**) The immune efficacy of the different risk groups was analyzed by the IMvigor210 dataset.

**Figure 8:**
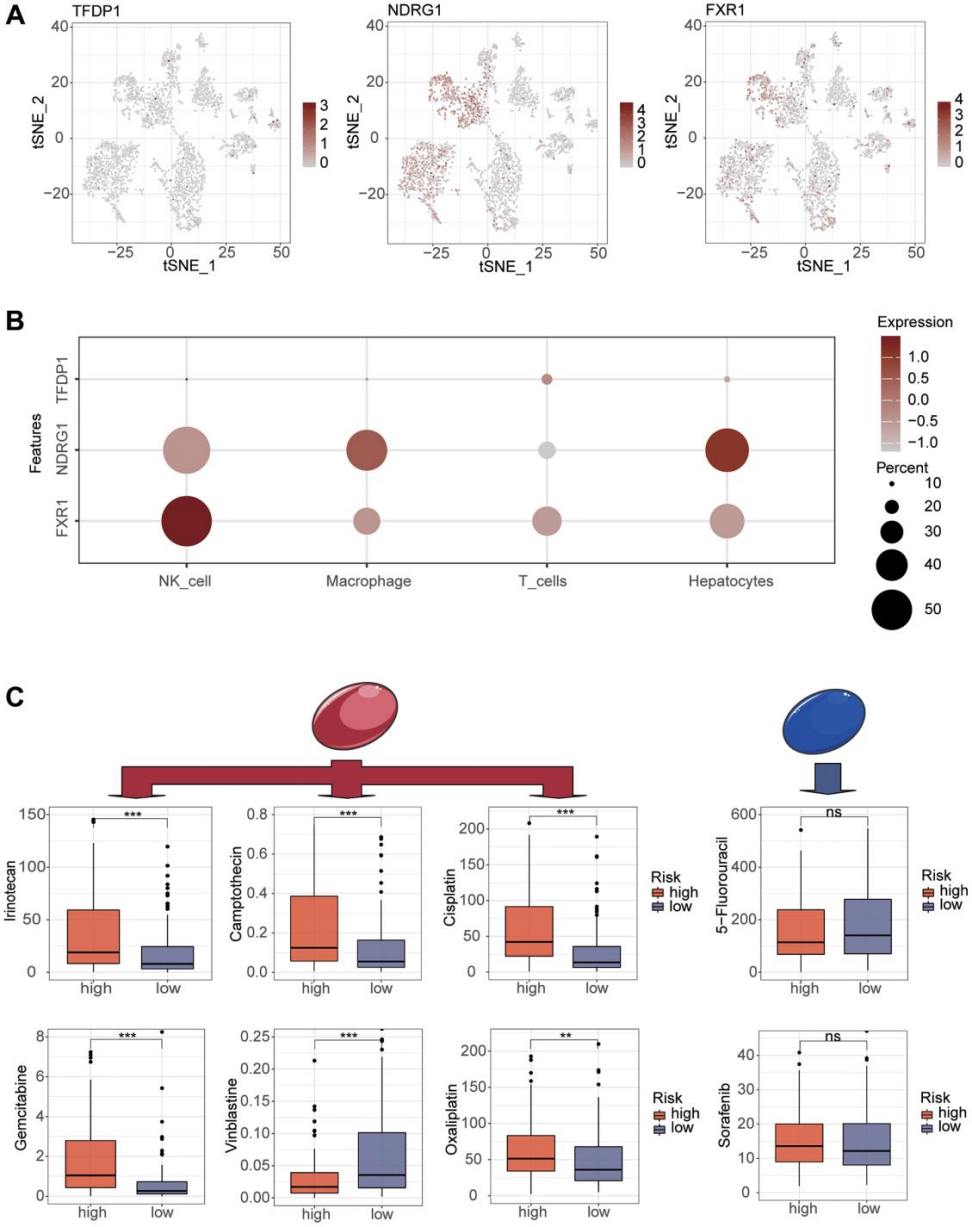
**Single-cell analysis of modeled key genes and drug sensitivity analysis of different risk groups.** (**A**, **B**) Single-cell analysis reveals the expression of modeling key genes in different cells. (**C**) Use the oncopredict package to explore chemotherapeutic agents that are significantly different between high and low risk groups.

To explore the relationship between risk score and immune cells, we investigated the abundance of more than 20 immune cells in the tumor microenvironment. By spearman correlation test, we found that risk score was significantly correlated with different immune cells. Among them, B cell memory, macrophage M0 and risk score were significantly positively correlated. B cell naive, plasma cells and risk scores were negatively correlated (Figure 7A). We then assessed whether there were differences in tumor microenvironment scores between the high and low risk groups. It can be seen that the immune score, stromal score and total score were significantly lower in the high-risk group than in the low-risk group (Figure 7B). There is growing evidence that increased expression of stem cell-associated biomarkers in tumor cells is highly correlated with drug resistance, cancer recurrence and tumor proliferation [21]. Our study found that this risk score was positively correlated with the tumor stemness score (Figure 7C). To understand the differences in immunotherapy between high and low risk groups, we predicted whether risk scores were associated with immunotherapy by MMR and immune checkpoint analysis. MMR were all positively correlated with risk scores, with MSH2 having the highest correlation (Figure 7D). The immune checkpoint analysis also found several indicators correlated with risk score, NRP1, TNFSF4, TNFSF15, TNFSF18, CD276, CD80 and HHLA2 were strongly correlated with risk score (*P* < 0.001). However, PD1, PDL1, and CTLA4 did not show correlation (Figure 7E). Subsequently, we verified with the dataset that risk score was a better predictor of the effectiveness of immunotherapy and that patients in the high-risk group had better immunotherapy outcomes (Figure 7F, 7G). To study the expression of key genes for constructing the model, we analyzed the expression of TFDP1, NDRG1 and FXR1 by single cell sequencing. Among them, TFDP1 expression was low in all four types of cells, while FXR1 was mainly expressed in NK cells. NDRG1 was highly expressed in hepatocellular carcinoma cells, macrophages and NK cells (Figure 8A, 8B). Subsequently, we investigated the sensitivity of high and low risk groups to different chemotherapeutic drugs. Interestingly, we found significant differences in drug sensitivity between the two groups for Camptothecin, Cisplatin, Gemcitabine, Irinotecan, Oxaliplatin and Vinblastine, but not for sorafenib and 5-Fluorouracil (Figure 8C).

### Experimental validation of key genes

**Figure 9:**
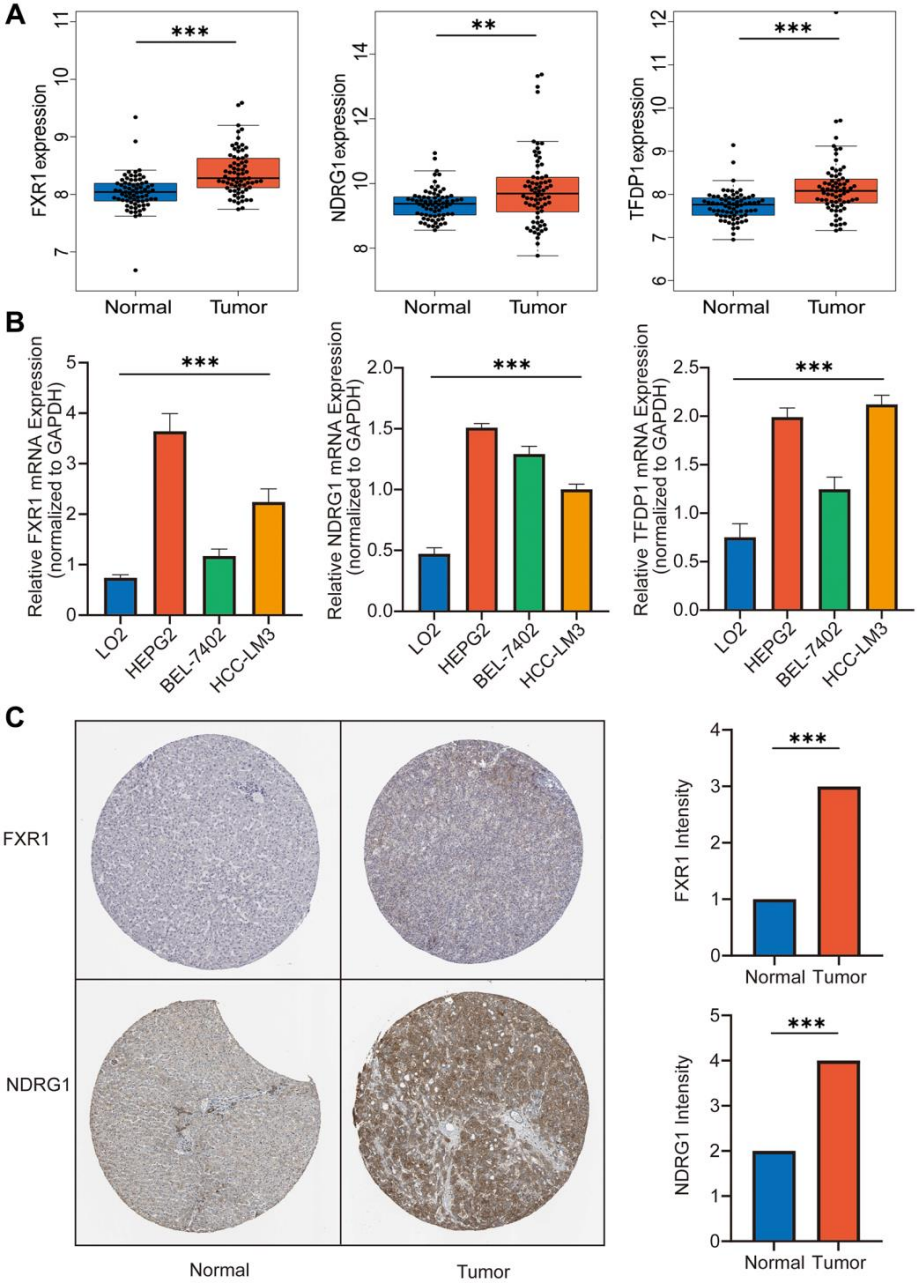
**Experiment *in vitro* and *in vivo*.** (**A**) Expression of FXR1, NDRG1 and TFDP1 was significantly higher in hepatocellular carcinoma tissues than in normal liver tissues by GEO dataset. (**B**) The expression of FXR1, NDRG1 and TFDP1 was found to be significantly higher in three types of hepatocellular carcinoma cells than in normal hepatocytes by RT-qPCR assay. (**C**) The expression of FXR1 and NDRG1 was found to be significantly higher in hepatocellular carcinoma tissues than in normal liver tissues using the HPA database.

We used the GSE39791 dataset for validation and could see that the expression of TFDP1, NDRG1 and FXR1 in liver cancer tissues was significantly higher than that in normal tissues (Figure 9A). Subsequently, we performed experimental validation using LO2 normal liver cells as well as the HEPG2, BEL-7402, and HCC-LM3 liver cancer cell lines. And the expression of the three key genes in different hepatocellular carcinoma cells was higher than that in normal hepatocytes (Figure 9B). Finally, IHC results showed that the expression of FXR1 and NDRG1 was significantly higher in liver cancer tissues than in normal tissues (Figure 9C).

## DISCUSSION

With the development of immunotherapy, new treatment options are bringing hope to liver cancer patients. Immunotherapy works by enhancing the body’s immune system to attack tumor cells, and unlike traditional treatment methods, it does not destroy normal cells, thus reducing many side effects [22]. One common immunotherapy method is the use of immune checkpoint inhibitors. Immune checkpoints are proteins that can help the body’s immune system recognize and attack tumor cells. However, certain tumor cells can use immune checkpoints to evade immune system attacks, leading to tumor growth and spread. The role of immune checkpoint inhibitors is to block these immune checkpoints, making tumor cells unable to escape immune attacks [23]. In the treatment of liver cancer, the use of immune checkpoint inhibitors has made some progress. Clinical studies have shown that some immune checkpoint inhibitors can enhance T cell immune responses, helping patients suppress tumor growth and spread, while also prolonging survival and improving quality of life [24].

This study first performed NMF classification using TCGA liver cancer transcriptome database, and the results showed that the samples were well divided into two clusters. KM analysis showed a significant difference in prognosis between the two clusters, and we then analyzed the potential mechanisms through GO and KEGG analyses. The MF analysis mainly enriched in protein serine/threonine kinase inhibitor activity, which has been found to be closely related to the occurrence and development of liver cancer in previous studies [25, 26]. Interesting KEGG pathway enrichment included Cell cycle, Cellular senescence, and HIF-1 signaling pathway. The disruption of the cell cycle is closely related to the occurrence and development of liver cancer, and HIF-1 abnormal activation plays an important role in the development of liver cancer, including promoting tumor cell growth, metabolism, and immune evasion [27, 28]. Considering that aging-related genes may be related to the prognosis of patients, we used the WGCNA algorithm to select an aging gene module that was correlated with patient prognosis. Then, we used the aging-related genes in this module to construct a lasso model, and the selected genes mainly included TFDP1, NDRG1, and FXR1. Previous studies have demonstrated that overexpression of TFDP1 can promote tumor cell growth, thereby accelerating the progression and deterioration of certain liver cancers [29]. NDRG1 can enhance the interaction between fibroblasts and tumor cells, leading to the development of hepatocellular carcinoma [30, 31]. FXR1 can promote the proliferation, invasion, and migration of hepatocellular carcinoma, and its action is mediated by Smad2/3 [32]. These studies provide additional evidence for the reliability of the genes we screened and are consistent with our predicted results. Afterwards, we performed survival analysis of the model using three datasets and found that patients in the high-risk group had significantly worse prognosis than those in the low-risk group. Additionally, the model demonstrated good prediction performance at 1, 2, and 3 years based on the ROC curves.

Furthermore, we used the Cibersort algorithm to explore the correlation between the model risk score and 21 immune cell types. We found a positive correlation between the risk score and B cell memory and macrophages M0, and a negative correlation with B cell naive and plasma cell. Previous studies have shown that a decrease in B cell naive is closely related to the occurrence and prognosis of liver cancer, which may be associated with immune escape and tolerance in the liver cancer microenvironment [33]. The quantity and function of plasma cells in liver cancer patients may also affect the immune status and treatment effectiveness [34]. Tumor stem cells are tumor cells with stem cell-like characteristics. Studies have shown that the existence and characteristics of liver cancer stem cells make liver cancer highly recurrent and resistant to treatment [35]. The stemness index is an indicator of the similarity between tumor cells and stem cells. Our study found a good correlation between this risk score and tumor stemness, demonstrating that the score can predict the degree of tumor stemness to a certain extent. This has important implications for subsequent treatment. Immunotherapy is an emerging cancer treatment method, but researchers have found that its efficacy varies greatly among different solid tumor patients [22, 23]. Therefore, we explored the correlation between this risk score and immunotherapy. MMR is a predictive indicator of immunotherapy, and our study found that the correlation between the risk score and indicators such as EPCAM, MLH1, MSH2, MSH6, and PMS2 was extremely high. Among them, the correlation between MSH2 and risk score was the highest, and studies have shown that mutations and abnormal expression of the MSH2 gene are closely related to the occurrence and development of various cancers [36]. Subsequently, we further verified the effectiveness of immunotherapy in patients with different risk scores using data sets, and found that patients with higher risk scores had better responses to immunotherapy. Single-cell sequencing technology can reveal the heterogeneity and evolutionary trajectories of different subclones within a tumor, which helps to deepen our understanding of the molecular mechanisms underlying cancer initiation and progression [37]. In order to further explore the expression patterns of key molecules in different cell types, we performed single-cell analysis using the GSE146115 dataset. We found that FXR1 had the highest expression level in NK cells. According to Zhang et al., natural killer cells (NK cells) play an important role in liver cancer immunotherapy, and their activity and infiltration level are closely related to the clinical prognosis of liver cancer [34]. NDRG1 was primarily expressed in liver cells. As for TFDP1, its expression levels were relatively low in various cell types, and it was mainly expressed in T cells. Studies have shown that T cells play an important role in tumor immunotherapy, and their immune surveillance and killing abilities are closely related to tumor progression and prognosis [22]. Subsequently, we performed drug sensitivity analysis and found that Camptothecin, Cisplatin, Gemcitabine, Irinotecan, Oxaliplatin and Vinblastine showed significant differences between the high risk and low risk groups. This has significant clinical implications for our diagnosis and treatment. Finally, through RT-qPCR experiments, we found that the expression levels of key genes involved in the construction of the model were higher in different liver cancer cells than in normal liver cells. IHC validation confirmed the expression patterns of these key genes in cancer tissue and normal tissue.

Certainly, our study has several limitations that need to be acknowledged. Firstly, we utilized multiple public databases for our joint analysis, but some of these databases lack clinical and immunotherapy data, which may result in certain omissions in our analysis. We plan to conduct further prospective studies to collect samples and data from our own hospital to conduct more in-depth research. Secondly, downstream mechanisms of the genes used to construct our model were not explored, which may lead to some bias in our prediction of targeted drugs. Further research is needed to address this issue.

In summary, our study has important clinical implications. The results obtained through the integration of bulk and single-cell sequencing data from multiple datasets are more reliable. The risk score constructed using WGCNA and LASSO can serve as a reliable and independent biomarker for predicting the prognosis of liver cancer patients. Single-cell sequencing analysis can help us further explore the expression patterns of hub genes in different cells. Functional enrichment analysis can assist in mechanism exploration and downstream analysis. In addition, our study explores the correlation between MMR and immune checkpoints through classification and model construction, which is helpful in assessing the effectiveness of immunotherapy. Furthermore, the difference in drug sensitivity between high and low risk groups determined by the risk model is useful for developing personalized chemotherapy regimens for patients.

## CONCLUSION

In summary, we have constructed an aging-related model, which we hope can serve as a reference for predicting patient survival and guiding liver cancer-related treatments.
